# Epigenetically regulated miR-449a enhances hepatitis B virus replication by targeting cAMP-responsive element binding protein 5 and modulating hepatocytes phenotype

**DOI:** 10.1038/srep25389

**Published:** 2016-05-03

**Authors:** Xiaoyong Zhang, Hongyan Liu, Zhanglian Xie, Wangyu Deng, Chunchen Wu, Bo Qin, Jinlin Hou, Mengji Lu

**Affiliations:** 1 Institute of Virology, University Hospital of Essen, University of Duisburg-Essen, Essen, Germany; 2 State Key Laboratory of Organ Failure Research, Guangdong Provincial Key Laboratory of Viral Hepatitis Research, Department of Infectious Diseases, Nanfang Hospital, Southern Medical University, Guangzhou, China

## Abstract

Cellular microRNAs (miRNAs) are able to influence hepatitis B virus (HBV) replication directly by binding to HBV transcripts or indirectly by targeting cellular factors. Here, we investigate the effect of epigenetically regulated miR-449a on HBV replication and the underlying mechanisms. miR-449a expression was lower in human hepatocellular carcinoma (HCC) cells than in primary hepatocytes and could be induced by trichostatin A. Ectopic miR-449a expression in HCC cells strongly enhanced HBV replication, transcription, progeny virions secretion, and antigen expression in a dose-dependent manner. miR-449a directly targeted cAMP-responsive element binding protein 5 (CREB5), which in turn induced the expression of farnesoid X receptor α (FXRα), a transcription factor that facilitates HBV replication. CREB5 knockdown and overexpression demonstrated that it is a negative regulator of HBV replication. Additionally, miR-449a overexpression inhibited proliferation, caused cell cycle arrest, and promoted HCC cell differentiation. The results indicated that epigenetically regulated miR-449a targets CREB5 to increase FXRα expression, thereby promoting HBV replication and gene expression. Our findings provide a new understanding of the role of miRNAs in HBV replication.

Hepatitis B virus (HBV) infection is a significant public health problem worldwide and is associated with hepatitis, liver cirrhosis, and hepatocellular carcinoma (HCC)^1^. Despite the availability of an efficient prophylactic vaccine, HBV infection remains highly prevalent, with approximately 240 million chronically infected patients and approximately one million deaths each year according to WHO estimations^2^. As the currently available therapies for chronic HBV infection are suboptimal and rarely cure patients completely^3^, there is an urgent need to elucidate the mechanisms underlying HBV replication and to identify novel molecular targets for HBV therapy. As major regulators of gene expression, microRNAs (miRNAs) play an important role in host–virus interactions^4^. Indeed, growing evidence indicates that many cellular miRNAs are involved in both the HBV life cycle and the development of HBV-associated liver diseases^5^.

miRNAs comprise a family of endogenous, conserved noncoding RNAs approximately 21–25 nucleotides in length that are involved in either translational arrest or RNA degradation *via* imperfect base pairing with the 3′-untranslated region (UTR) or coding region of the target transcript^6^. Briefly, miRNAs are transcribed from the host genome and generated by Drosha- and Dicer-mediated enzymatic cleavage^7^. Epigenetic modifications, such as DNA methylation and histone acetylation, have been demonstrated to affect the expression of a set of miRNAs^8^, and interestingly, these miRNAs can also affect the expression of epigenetically regulated genes by targeting key enzymes responsible for epigenetic reactions^9^. Accordingly, these miRNAs related to epigenetic regulation have been defined as “epi-miRNAs”, the aberrant expression of which is often related to the development or progression of human cancer^10^.

Many cellular miRNAs modulate HBV replication by either directly binding to HBV transcripts or targeting cellular transcription factors required for HBV gene expression^11^. For example, miR-125a-5p^12^ and miR-1231^13^ directly target HBV mRNAs, reducing viral replication and gene expression. miR-130a suppresses HBV replication by targeting two major metabolic regulators, PGC1α and PPARγ, both of which can potently stimulate HBV replication^14^. A number of studies have identified differentially expressed epi-miRNAs in HCC tissues versus normal liver tissues or HBV-infected cells versus control cells^15^. Previously, we showed that miR-1, an epi-miRNA linked to the epigenetic regulation of HCC^16^, indirectly regulates HBV replication by targeting histone deacetylase 4 (HDAC4) and E2F transcription factor 5, leading to increased HBV replication^17^.

Recently, miR-449a has been reported to be downregulated in several cancer cell lines and solid tumors, including HCC^18^, prostate cancer^19^, gastric cancer^20^, colorectal cancer^21^, and lung cancer^22^. As a tumor-suppressive miRNA, miR-449a inhibits cell growth and proliferation in a retinoblastoma (Rb)-dependent manner by directly targeting key factors involved in cell cycle progression, such as HDAC1^19^, cyclin D1^23^, CDC25A^24^, cyclin-dependent kinase 6 (CDK6)^20^ and E2F transcription factor 1 (E2F1)^24^. Interestingly, HDAC1-3 upregulation reduces the expression of miR-449a in HCC cell lines, whereas miR-449a overexpression reduces the expression of its target c-MET, decreases the phosphorylation of extracellular signal-regulated kinases 1 and 2 (ERK1/2), and inhibits the proliferation of HCC cells^18^. Notably, both the HDAC1 and ERK pathways, which are targeted by miR-449a, were previously reported to be involved in regulating HBV replication^25^,^26^.

However, very few studies to date have investigated the molecular mechanisms of interactions between epi-miRNAs and HBV infection. Therefore, this study aims to examine the effect of miR-449a regulation on HBV and to explore the underlying molecular mechanisms.

## Results

### Upregulation of HBV replication and miR-449a expression by the HDAC inhibitor TSA in HCC cells

**Figure 1:**
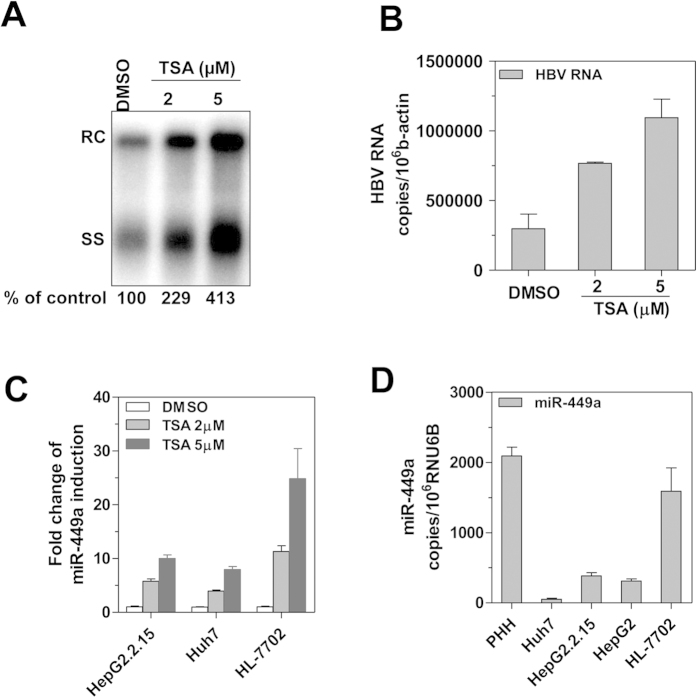
Regulation of HBV replication and miR-449a expression by the HDAC inhibitor TSA. (**A**) HBV plasmid pSM2-transfected Huh7 cells were treated with 2 or 5 μM of TSA for 2 days, and HBV replication was determined by southern blotting. The positions of relaxed circular (RC) and single-stranded (SS) DNAs are indicated. (**B**) HBV RNA levels in pSM2-transfected Huh7 cells were determined by real-time RT-PCR. (**C**) miR-449a induction in HepG2.2.15, Huh7 and HL-7702 cells after TSA treatment for 24 hours was measured by real-time RT-PCR. (**D**) Baseline miR-449a expression in PHH, Huh7, HepG2.2.15, HepG2, and HL-7702 cells was quantified by real-time RT-PCR.

We previously reported that trichostatin A (TSA), a potent HDAC inhibitor that increases histone acetylation, could enhance HBV replication in HBV-stably transfected HepG2.2.15 cells^17^. In the present study, TSA treatment in Huh7 cells transiently transfected with the replication-competent HBV plasmid pSM2 led to an increase of HBV replicative intermediates (Fig. 1A) and transcripts (Fig. 1B). Similarly, TSA treatment also increased intracellular HBV DNA and HBsAg levels in a non-tumor hepatocytes cell line HL-7702 cells (Suppl. Fig. 1A). These results are consistent with a previous observation in cell-based replication systems and patient liver samples that HBV replication is regulated by the acetylation status of H3/H4 histones bound to the viral genome^25^. Similar to cell lines from testicular cancer^27^, we also observed an approximately 4–30-fold induction of miR-449a expression in TSA-treated HepG2.2.15 and Huh7 cells, as well as in HL-7702 cells, respectively (Fig. 1C). Further, the baseline expression of miR-449a in the HepG2.215 cells and its parent cell line HepG2 were similar, and these cells including huh7, expressed less miR-449a than in primary human hepatocytes (PHH) and HL-7702 (Fig. 1D), suggesting that miR-449a may be epigenetically silenced during the malignant transformation of hepatocytes.

### Dose-dependent enhancement of HBV replication, transcription and gene expression by miR-449a

**Figure 2:**
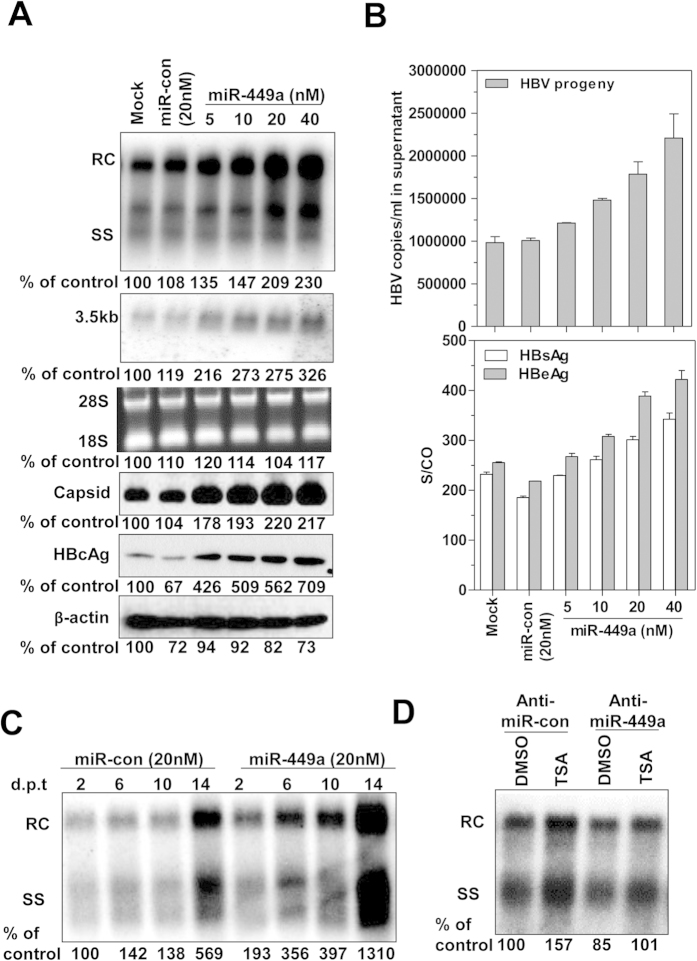
Upregulation of HBV replication, transcription, and gene expression by miR-449a. (**A**) HepG2.2.15 cells were transfected with miR-con or miR-449a at the indicated concentrations. Cell lysates and culture supernatants were harvested at day 4 after transfection. HBV replication and transcription were detected by Southern blotting (upper panel) and northern blotting (middle panel), respectively. Intracellular HBV capsid and HBcAg were measured by western blotting (bottom panel). β-actin and 28S and 18S RNAs were used as loading controls. (**B**) HBV progeny DNA in cell culture supernatants was quantified by real-time PCR (upper panel). The levels of secreted HBsAg and HBeAg in culture media were measured by the CMIA test (bottom panel). (**C**) HBV replication was detected by Southern blotting at the indicated time points after transfection. (**D**) Huh7 cells were transfected with 50 nM of anti-miR-449a or anti-miR-con for 2 days, then treated with 2 μM of TSA for 24 hours. HBV replication was determined by Southern blotting.

Considering that the miR-449a-targeted HDAC1 and ERK pathways are involved in regulating HBV replication, we tested the effect of miR-449a on HBV. HepG2.2.15 cells were transfected with a miR-449a mimic at concentrations ranging from 5 to 40 nM, and HBV replication and gene expression were analyzed after 4 days. Compared with control miRNA, miR-449a increased HBV replicative intermediates and HBV 3.5-kb RNA transcripts, intracellular HBcAg expression, and HBV capsid formation (Fig. 2A), as well as the number of HBV progeny and HBsAg and HBeAg concentrations in culture supernatants (Fig. 2B) in a dose-dependent manner. Moreover, the upregulation of HBV replication by miR-449a was detectable beginning on day 2 after transfection, increased with time, and was maintained at least up to day 14 (Fig. 2C). Consistently, co-transfection with miR-449a and pSM2 in Huh7 cells resulted in enhanced HBV replication and HBsAg and HBeAg expression (Suppl. Fig. 1B). Compared with miR-1, the effect of miR-449a on HBV replication was stronger, but no synergistic effect was observed in HepG2.2.15 cells (Suppl. Fig. 1C). Due to the relatively lower expression of miR-449a in Huh7 cells, transfected with the miR-449a inhibitor anti-miR-449a did not affect baseline HBV replication significantly, but it could partially block the enhancing effect of TSA on HBV replication (Fig. 2D). Additionally, neutralizing endogenous miR-449a by anti-miR-449a in HL-7702 cells was able to reduce HBV replication and HBsAg expression (Suppl. Fig. 1D).

### FXRα upregulation mediates the increased HBV core promoter activity and enhanced HBV replication caused by miR-449a

**Figure 3:**
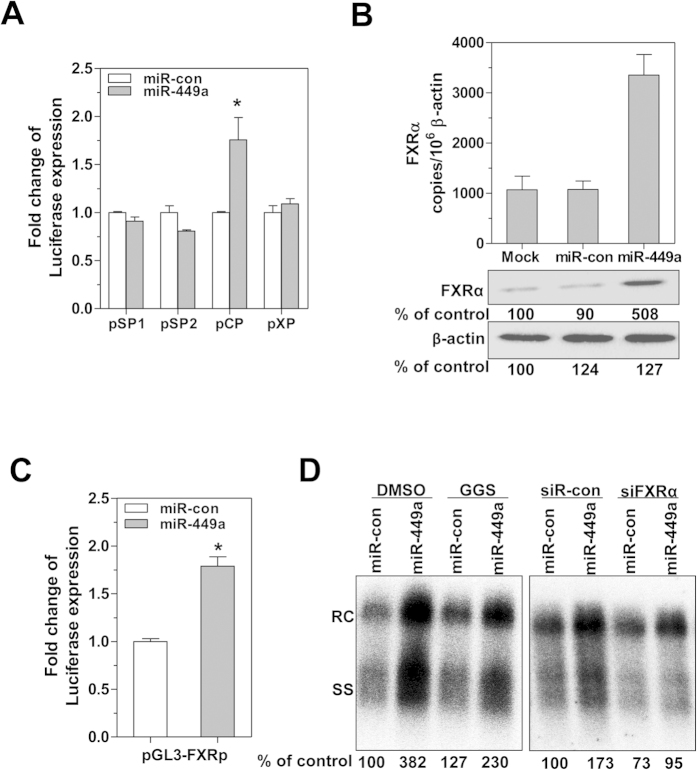
MiR-449a transactivates the HBV core promoter by upregulating FXRα expression. (**A**) Luciferase reporters containing the HBV promoter regions pSP1, pSP2, pCP, and XP were co-transfected with miRNA into HepG2.2.15 cells; assays for luciferase activity were performed at 48 hours. The relative luciferase expression was expressed as the ratio of miR-449a- to miR-con-transfected samples. (**B**) FXRα mRNA and protein levels in HepG2.2.15 cells were assessed by real-time RT-PCR and western blotting at day 4 following transfection with miR-449a (20 nM). (**C**) Luciferase reporters containing the FXRα promoter region pGL3-FXRα-promoter were co-transfected with miR-449a into HepG2.2.15 cells; assays for luciferase activity were performed at 48 hours. (**D**) HepG2.2.15 cells were transfected with 20 nM miR-449a and then treated with the FXRα antagonist GGS (10 μM) or co-transfected with 20 nM of FXRα-specific siRNA for 4 days. HBV replication was detected by Southern blotting. *P < 0.05.

A direct interaction between a miRNA and its target mRNA requires the presence of a complementary seed sequence in the target miRNA^7^. However, bioinformatics analysis using the HBV genomic sequences available in GenBank did not identify a sequence complementary to the miR-449a seed sequence (CACUGCC) (data not shown). Thus, to elucidate the molecular mechanisms of the effect of miR-449a on HBV replication, the global gene expression profile of HepG2.2.15 cells after miR-449a transfection was determined by microarray analysis. A heatmap showed that similar to miR-1, miR-449a transfection increased farnesoid X receptor α (FXRα) expression (Suppl. Fig. 2A). FXRα, also called NR1H4 (nuclear receptor subfamily 1, group H, member 4), is a transcription factor that binds to two motifs within the HBV enhancer II and core promoter regions to promote HBV transcription and replication^28^. Consistently, miR-449a increased transcription of the HBV core promoter to approximately 1.8-fold but had no significant effect on HBV SP1, SP2, and X promoter activity (Fig. 3A). The upregulation of FXRα by miR-449a was further verified by real-time RT-PCR and western blot analysis (Fig. 3B). Furthermore, FXRα promoter activity was found to be enhanced after miR-449a transfection (Fig. 3C); both FXRα promoter activity and expression were also induced by TSA treatment in HCC cells (Suppl. Fig. 2A,B). However, the enhancement of HBV replication by miR-449 was partially blocked by the natural FXRα antagonist guggulsterone (GGS) or a validated siRNA targeting FXRα (Fig. 3D). Taken together, miR-449a enhances HBV replication and gene expression at least partly by upregulating FXRα expression.

### CREB5 is a negative regulator of HBV replication and a direct target of miR-449a

**Figure 4:**
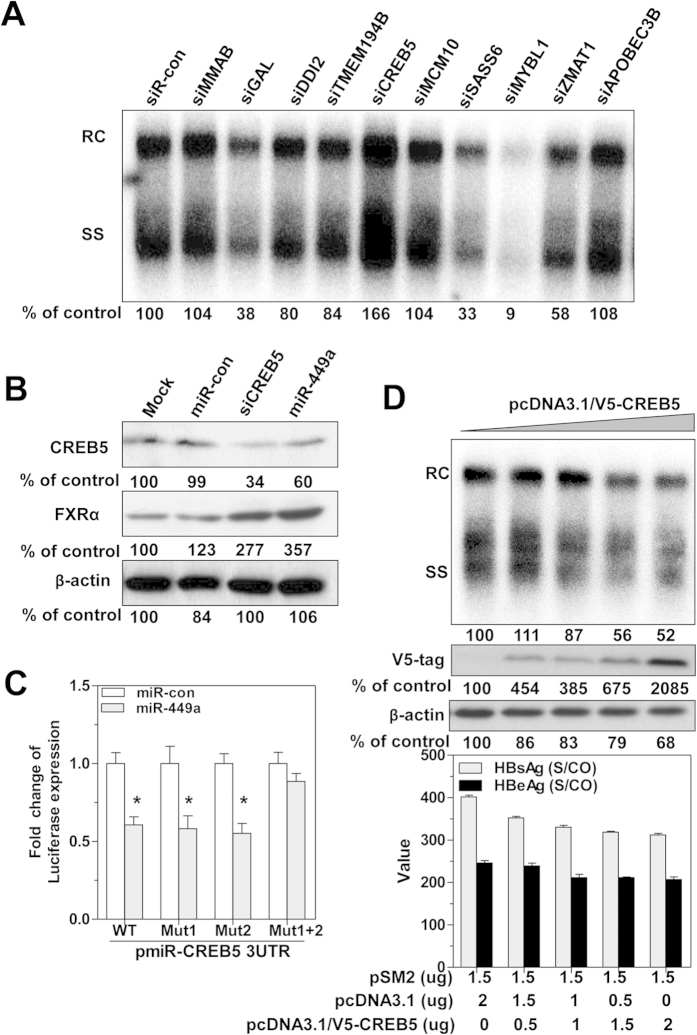
CREB5 is directly targeted by miR-449a and is involved in the control of HBV replication. **(A)** HepG2.2.15 cells were transfected with 20 nM of different siRNAs targeting *MMAB, GAL, DDI2, TMEM194B, CREB5, MCM10, SASS6, MYBL1, ZMAT1*, and *APOBEC3B* for 4 days. HBV replication was detected by Southern blotting. (**B**) Western blot analysis of CREB5 and FXRα protein expression in HepG2.2.15 cells transfected with miR-449a or siRNA specific for CREB5 at 20 nM for 3 days. (**C**) Wildtype and mutated pmiR-CREB5-3UTR luciferase reporters (100 ng each) were co-transfected with 20 nM of miR-449a in Huh7 cells, luciferase activity was assayed at 48 h. The fold change of luciferase expression expressed as the ratio of miR-449a- to miR-con-transfected samples. (**D**) The CREB5 expression vector pcDNA3.1/V5-CREB5 was co-transfected in Huh7 cells with pSM2 at the indicated concentrations for 4 days. HBV replication was detected by Southern blotting, and CREB5 expression was determined by V5-tag western blotting. The levels of secreted HBsAg and HBeAg in culture media were measured by the CMIA test. *P < 0.05.

We next addressed whether miR-449a-targeted genes are involved in regulating HBV replication. Microarray analysis identified 14 genes with expression levels that decreased by more than 2-fold after miR-449a transfection (Suppl. Fig. 3A), and ten (*MMAB, GAL, DDI2, TMEM194B, CREB5, MCM10, SASS6, MYBL1, ZMAT1*, and *APOBEC3B*) were selected for validation. Pre-designed siRNAs targeting these genes were transfected into HepG2.2.15 cells to assess their effect on HBV replication. siRNA targeting Cyclic AMP-Responsive Element-Binding Protein 5 (CREB5) exerted the strongest enhancement effect on HBV replication; except for a *MYBL1* siRNA, which inhibited viral replication, siRNAs targeting the other genes had no significant effect (Fig. 4A).

The CREB5 gene belongs to the CRE (cAMP response element)-binding protein family: the protein specifically binds to CRE as a homodimer or heterodimer with c-Jun or CRE-BP1 to function as a CRE-dependent transactivator^29^. Using miRNA target prediction softwares (TargetScan, MiRanda, DIANAmT, miRWalk, and Pictar5), CREB5 was identified as a potential target of miR-449a, with two evolutionarily conserved complementary seed sequences (nt 3259–3265, nt 3317–3323) in the 3′UTR of its mRNA (Suppl. Fig. 3B). Western blot analysis demonstrated that CREB5 expression was reduced by CREB5 siRNA and miR-449a transfection, whereas FXRα expression was increased after transfection (Fig. 4B). To determine whether CREB5 is directly targeted by miR-449a, four luciferase reporter plasmids were constructed bearing the wildtype and three mutant versions of the CREB5 3′UTR. Co-transfection with miR-449a reduced the luciferase activity of the wildtype CREB5 3′UTR reporter, whereas the 3′UTR mutant construct containing two mutation sites was fully protected from miR-449a-mediated repression (Fig. 4C). Furthermore, CREB5 overexpression, which was measured by V5-tag immunoblot analysis, led to decreased HBV replication and HBsAg and HBeAg production in Huh7 cells in a dose-dependent manner (Fig. 4D). These findings show that CREB5 is a direct target of miR-449a and negatively regulates HBV replication.

### miR-449a inhibits cell cycle transition and proliferation in hepatoma cells and promotes differentiation

**Figure 5:**
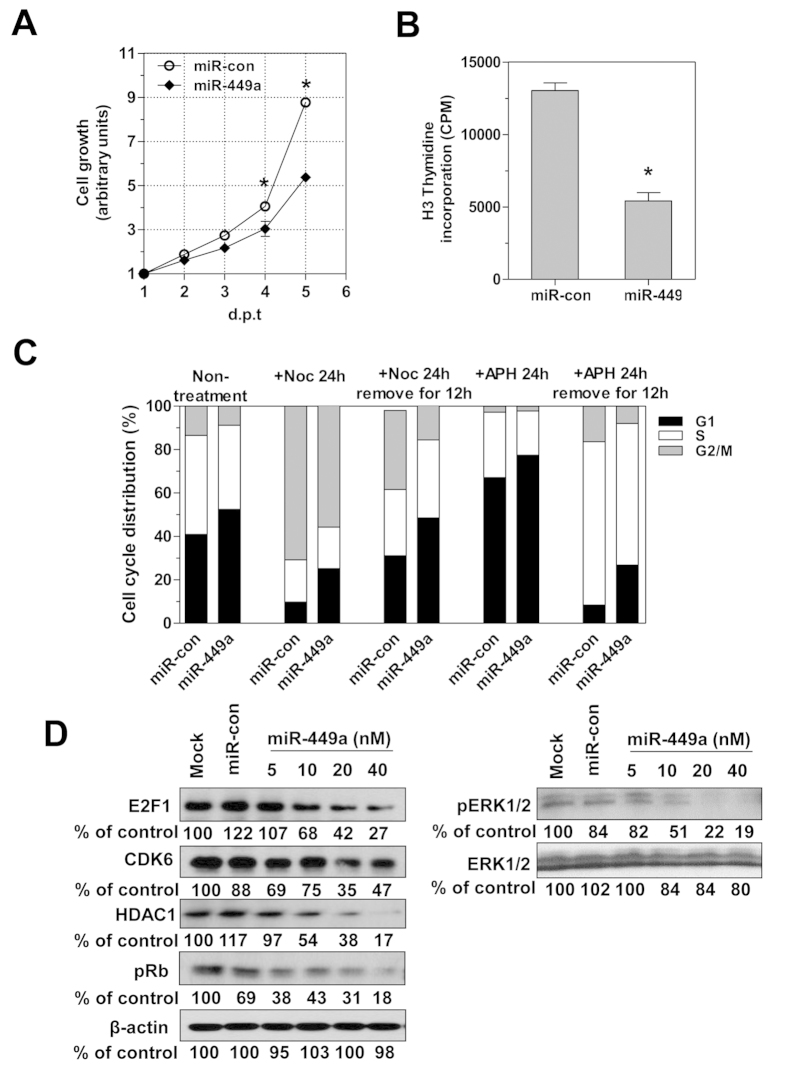
MiR-449a inhibits cell proliferation and arrests the cell cycle by targeting multiple genes. HepG2.2.15 cells were transfected with 20 nM miR-449a. After 24 h, the cells were split at a density of 10,000/well to perform the following experiments. (**A**) Cells were distributed into 96-well plates, and cell growth was measured every 24 h for 5 days using the WST-1 assay. (**B**) Cells were serum-starved overnight for 48 hours, followed by the addition of serum and ^3^H-thymidine; incorporation of ^3^H-thymidine into cellular DNA after 4 h was measured using a scintillation counter. (**C**) Cells were treated with nocodazole (100 nM), aphidicolin (4 μg/ml) or medium control for 24 h. The distribution of cells in the cell cycle phases G1, S, and G2/M was assessed by flow cytometry using propidium iodide staining, and the percentage of cells in the different phases of the cell cycle is shown. Each experiment was performed in triplicate with two different batches of transfected cells. (**D**) HepG2.2.15 cells were transfected with different concentrations of miR-449a for 4 days. E2F1, CDK6, HDAC1, ERK1/2, phosphorylated-Rb and-ERK1/2 protein levels were measured by western blotting with specific antibodies and normalized to β-actin as a loading control. *P < 0.05.

**Figure 6:**
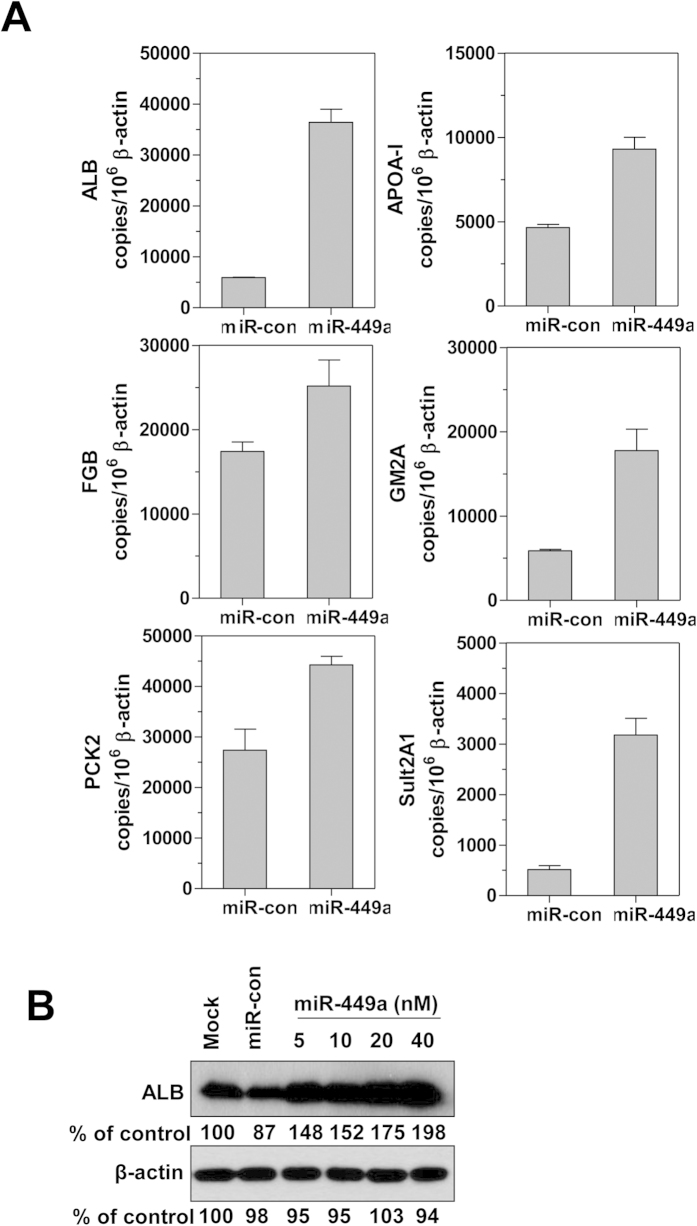
miR-449a promotes liver-specific gene expression in HepG2.2.15 cells. (**A**) HepG2.2.15 cells were transfected with 20 nM of miR-449a or miR-con for 4 days. The expression levels of ALB, APOA-I, FGB, GM2A, PCK2, and Sult2A were determined by real-time RT-PCR; the results are presented as copies per 10^6^ β-actin transcripts. (**B**) HepG2.2.15 cells were transfected with the indicated concentrations of miR-449a for 4 days. ALB protein levels were assessed by western blotting with specific antibodies and normalized to β-actin as a loading control.

To further understand the mechanism involved in HBV upregulation, we utilized GSEA analysis of expression profiles to investigate the biological effect of miR-449a. The results showed that the downregulation of predicted target gene set of miR-449a family and cell cycle-related gene set was enriched in miR-449a transfected HepG2.2.15 cells, while a cluster of liver-specific genes with enhanced expression was also found (Suppl. Fig. 4A–C). Consistently, cell growth (Fig. 5A) and DNA synthesis (Fig. 5B) of HepG2.2.15 cells were decreased by miR-449a transfection. The effects of aphidicolin and nocodazole, two cell cycle inhibitors that synchronize cells at the G1 and G2/M phase, respectively, were then examined in miR-449a transfected HepG2.2.15 cells. A cell cycle distribution analysis showed that miR-449a led to cell cycle arrest at the G1 phase and increased the cell population in this phase, even after the inhibitors were removed (Fig. 5C). Similar results were obtained within Huh7 cells (Suppl. Fig. 5A–C). Western blot analysis of whole-cell extracts showed that expression of the previously reported targets E2F1, CDK6, and HDAC1 was reduced by miR-449a in a dose-dependent manner (Fig. 5D); sequential de-phosphorylation of Rb and ERK during cell cycle arrest was also mediated by miR-449a transfection (Fig. 5D). In addition, the mRNA levels of six representative genes for hepatocyte differentiation, albumin (ALB), apolipoprotein A1 (APOA-I), fibrogen β (FGB), GM2 ganglioside activator (GM2A), phosphoenolpyruvate carboxykinase 2 (PCK2), and sulfotransferase 2A (Sult2A1), were increased significantly at day 4 after miR-449a transfection (Fig. 6A). Increased ALB protein expression was also determined by western blot analysis (Fig. 6B). Taken together, miR-449a targets multiple genes to inhibit cell growth and promote the differentiation of hepatoma cells, which is generally beneficial for HBV replication.

### FXRα up-regulation, cell cycle arrest, and differentiation play roles together for enhancement of HBV replication by epi-miRNA in hepatoma cells

**Figure 7:**
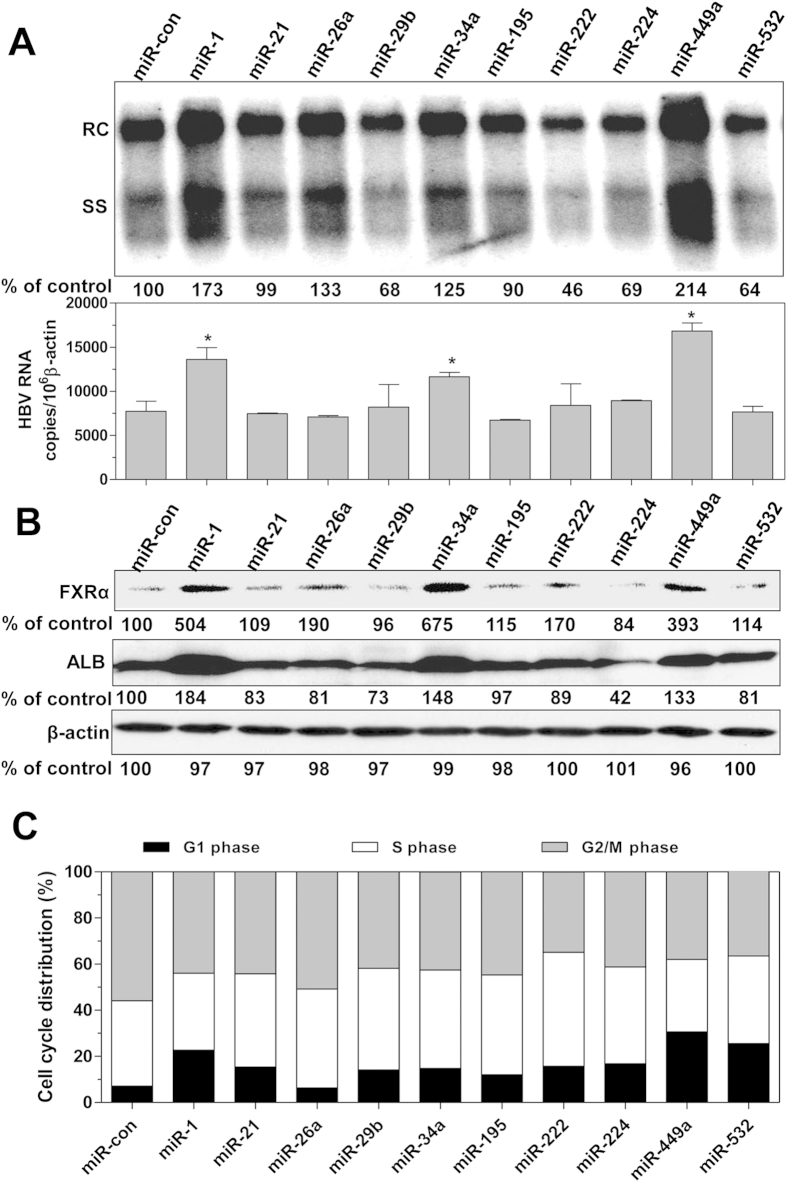
Regulation of HBV replication and transcription, FXRα and ALB expression, and cell cycle progression by HCC-related miRNAs. HepG2.2.15 cells were transfected with different miRNA mimics at 20 nM and then cultured for 4 days. (**A**) HBV replication and transcription were determined by Southern blotting (upper panel) and real-time RT-PCR (bottom panel), respectively. (**B**) FXRα and ALB expression was determined by western blotting. (**C**) miRNA-transfected HepG2.2.15 cells were treated with nocodazole (100 nM) for 24 hours, and the distribution of cells in cell cycle phases G1, S, and G2/M was assessed by flow cytometry using propidium iodide staining. *P < 0.05.

Based on the above findings, a question was raised with regard to whether other HCC-related epi-miRNAs exert an effect on HBV replication that is similar to miR-449a. Eight miRNA mimics in addition to miR-1 and miR-449a were selected according to a previous report^30^ about altered expression levels in HCC tissues and transfected into HepG2.215 cells. Interestingly, we found that miR-34a, which shares the same seed sequence as miR-449a, had an enhancing effect on HBV replication and transcription (Fig. 7A), with the concomitant upregulation of HBsAg and HBeAg (Suppl. Fig. 6A). In addition, FXRα and ALB expression were increased at both the protein and mRNA levels (Fig. 7B, Suppl. Fig. 6B). In contrast, the effect of miR-34a on HBV replication was relatively weak compared to miR-1 and miR-449a. A possible explanation might be the fact that miR-34a has a rather small effect on cell cycle and proliferation (Fig. 7C, Suppl. Fig. 5C). Although another candidate miRNA, miR-532, was able to arrest the cell cycle and significantly inhibit proliferation (Fig. 7C, Suppl. Fig. 6C), it did not have an effect on FXRα and ALB expression or on HBV replication (Fig. 7A,B, Suppl. Fig. 6B). However, miR-532 did increase HBsAg expression in HepG2.2.15 cells (Suppl. Fig. 6A). Based on these results, we speculate that a combination of FXRα upregulation, cell cycle arrest, and cell differentiation in hepatoma cells may be required for the enhancement of HBV replication and gene expression by epi-miRNAs.

## Discussion

In the current study, we identified and explored the role of miR-449a in the regulation of HBV replication. By dissecting the relationship between histone deacetylation and miR-449a regulation, we identified CREB5 as a target gene of miR-449a in HCC cells. CREB5 knockdown increased FXRα expression and led to the transcriptional activation of HBV. Moreover, miR-449a arrested the cell cycle at G1 phase and induced HCC differentiation. Our results demonstrated that epi-miRNA miR-449a acts as a novel linker between histone deacetylation and HBV replication, creating a favorable environment for HBV replication in hepatocytes.

Epigenetic modifications such as histone acetylation or deacetylation regulate HBV transcriptional activity and replication^31^. In a cell-based replication system as well as in the liver of chronic HBV-infected patients, chromatin immunoprecipitation assay revealed the recruitment of histone deacetylase HDAC1 to HBV covalently closed circular DNA (cccDNA). Here, we confirm that TSA is able to enhance HBV replication and transcription in HBV plasmid transiently transfected into Huh7 cells. Moreover, we observe that TSA treatment increases FXRα promoter activity and mRNA expression (Suppl. Fig. 2A,B), and we find that miR-449a expression is induced by TSA treatment and that its overexpression enhances HBV replication, transcription and gene expression. Considering that HDAC1 is a direct target of miR-449a, we speculate that TSA might induce miR-449a expression to exert its inhibitory effect on HDAC1 expression, thereby enhancing HBV replication and gene expression in an FXRα-dependent manner. We also observed that miR-449a transfection consistently up-regulates HBeAg expression and capsid formation in a HBV cccDNA model cell line, HepDES19^32^, leading to the enhancement of HBV transcription and replication from the cccDNA template (Suppl. Fig. 7A,B).

HBV transcription from cccDNA is tightly regulated by a number of liver-enriched transcription factors and nuclear receptors *via* the recognition of HBV promoter/enhancer elements^33^. Many miRNAs share a common pattern of targeting transcription factors to regulate HBV replication. For example, miR-372 and -373 promote HBV gene expression through a pathway involving nuclear factor I/B^34^, and miR-15b enhances HBV enhancer I activity by targeting hepatocyte nuclear factor 1α, a negative regulator of HBV enhancer I^35^. In contrast, miR-141 targets peroxisome proliferator-activated receptor α to suppress HBV expression and replication in HepG2 cells^36^. Previously, we showed that miR-1 increased the transcriptional activity of the HBV core promoter by augmenting FXRα expression^17^. Interestingly, miR-449a transfection also resulted in an increase in FXRα promoter activity and mRNA and protein expression. Furthermore, CREB5 was found to be directly targeted by miR-449a, and CREB5 knockdown increased FXRα expression. According to our results, the regulatory effect of miR-449a on HBV replication might be dependent on CREB5 regulation and FXRα expression. In addition, previous studies have shown that the HBV enhancer I and S promoter direct the liver-specific transcription of viral genes and contain a cAMP-response element sequence. CREB1 is able to bind to these elements and is required for HBV replication^37^,^38^. We observed that CREB5 overexpression inhibited HBV replication and gene expression. However, whether CREB5 competitively inhibits the CREB1/HBV interaction needs to be further investigated.

In addition to transcription factors, intracellular signaling pathways and hepatocyte phenotypes also regulate HBV replication and gene expression. Previously, we demonstrated that the toll-like receptor 2 and 4-mediated activation of intracellular MAPK-ERK and PI3k/Akt pathways in hepatocytes is required for their ability to suppress HBV replication^26^,^39^. Interestingly, both HDAC inhibition and miR-449 overexpression in HCC cells led to reduced c-MET expression, increased apoptosis, and decreased proliferation. ERK 1/2, two downstream effectors of c-MET, consistently display reduced phosphorylation and activation^18^, and we also confirmed that miR-449a is able to reduce ERK1/2 phosphorylation. Thus, there is a possibility that miR-449a enhances HBV replication through a blockade of the MAPK-ERK pathway. Moreover, analysis of the cellular gene expression profile revealed that miR-449a overexpression results in the downregulation of a set of cell cycle-related genes and the upregulation of multiple genes related to a highly differentiated hepatocyte phenotype. In general, HBV replication is associated with the cell cycle and differentiation status of hepatocytes. Therefore, modification of the HCC cell phenotype *via* miR-449a overexpression could also contribute to the upregulation of HBV replication and gene expression.

Many studies have shown that epi-miRNAs play a critical role in HBV-associated HCC development and, can function as oncogenes or tumor suppressor genes depending on the cellular function of the target genes^40^. The HBx protein has been implicated as a potential trigger of the epigenetic modifications of miRNAs during hepatocarcinogenesis^41^. It was found that miR-152 was downregulated by HBx protein, causing an upregulation of its target DNA methyltransferase 1 expression and subsequently methylation of tumor suppressor genes to induce HCC^42^. In the present study, we found that miR-449a expression in HCC cell lines was low and could be regulated by HDAC inhibitor, consistent with the previous reports that miR-449 expression was suppressed in liver cancerous tissue^43^, likely due to the epigenetic silencing of miR-449a by HDAC1-3^18^. Moreover, the dual function of miR-449a on HBV replication and cell proliferation could partially explain the lower levels of HBV replication and gene expression in the cancerous tissue than adjacent normal liver tissue^44^,^45^.

Taken together, our data indicated that miRNAs may be considered as one of the most important cellular regulatory factors involved in regulating the expression of HBV genes as well as cellular genes and signaling pathways that play critical roles in viral replication and pathogenesis. Concerning the similar mechanisms of miR-1 and miR-449a in HBV replication, epi-miRNAs might have a general effect on HBV infection. Nonetheless, further research is needed to clarify the mechanisms by which host miRNA expression is altered during HBV infection and the role of miRNA alterations in cellular function and liver disease progression.

## Material and Methods

### Reagents

All siRNAs, miRNAs and its inhibitors used in the present study are listed in Supplementary Table 1. The class I HDAC inhibitor TSA, FXRα antagonist guggulsterone (GGS), and cell cycle synchronization chemicals aphidicolin and nocodazole were purchased from Sigma-Aldrich (Steinheim, Germany).

### Cell culture and transfection

Human hepatoma cell lines HepG2 and Huh7, and normal hepatocytes cell line HL-7702 were grown in Dulbecco’s modified Eagle’s medium supplemented with 10% fetal bovine serum, 100 U/ml penicillin and 100 μg/ml streptomycin and maintained at 37 °C in a humidified 5% CO_2_ atmosphere. HepG2.2.15 cells with integrated dimers of the HBV genome were cultured with 500 μg/ml of G418 (Sigma-Aldrich). Primary human hepatocytes were isolated from 3 liver transplantation donors by perfusion and then cultured, as described previously^26^. Plasmids, miRNAs and siRNAs were transfected into cells at the indicated concentrations using Lipofectamine 2000 (Invitrogen, Carlsbad, CA) according to the manufacturer’s instructions.

### Analysis of HBV replication and gene expression

HBV replicative intermediates from intracellular core particles and HBV transcripts were extracted from hepatoma cell lines and detected by Southern and northern blotting, respectively, according to previously published protocols^17^. HBV progeny DNA was extracted from cell culture supernatants using the QiAamp DNA Blood Mini kit (Qiagen, Hilden, Germany) and quantified by real-time PCR as described^17^. HBV RNA in cells was also detected using quantitative real-time RT-PCR (the primers sequences are listed in Supplemental Table 2). A monoclonal antibody (clone 10E11, Santa Cruz Biotechnology, CA) was used to detect intracellular HBcAg expression and HBV capsid by western blotting, as described below. The levels of HBsAg and HBeAg in culture supernatants were determined using Architect system and HBsAg and HBeAg CMIA kits (Abbott Laboratories, Wiesbaden-Delkenheim, Germany) according to the manufacturer’s instructions.

### Western blot analysis

Protein samples were subjected to SDS-PAGE, blotted and then probed with primary antibodies against the following: ALB, CDK6, E2F1, HDAC1, ERK1/2, phosphorylated-Rb and -ERK1/2 (Cell Signaling Technology, Danvers, MA); CREB5 and FXRα (R&D System, Minneapolis, MN); and β-actin (Sigma-Aldrich). Protein bands were visualized using ECL Plus Western blotting detection reagents (Amersham Biosciences, Buckinghamshire, UK). The grey scales of protein or nucleic acid bands were analyzed by Image-Pro Plus software (Media Cybernetics Inc, Silver Spring, MD) and the values were presented below each of the blots as the percentage of control samples.

### Real-time RT-PCR assay

Total RNA, including miRNAs, was extracted with TRIzol (Invitrogen) and cleaned with the RNeasy Mini kit or miRNeasy Mini kit, which involved digestion with DNase Set (Qiagen). The expression of miR-449a and different cellular genes was determined by quantification of specific targets using commercial Quantitect Primer Assays (Qiagen, primer sequences not available). Real-time RT-PCR was performed using a one-step method with 100 ng of total RNA and a QuantiFast SYBR Green RT-PCR Kit (Qiagen) or a two-step method using a miScript SYBR Green PCR Kit and a Light CyclerTM (Roche Diagnostics, Mannheim, Germany).The expression levels of cellular genes and miRNA are presented as values normalized against 10^6^ copies of β-actin transcripts and U6 snRNA (RNU6B), respectively.

### Vector construction and luciferase reporter assay

The HBV replication-competent plasmid pSM2 harboring a head-to-tail tandem dimer of the HBV genome was provided by Dr. Hans Will (Heinrich-Pette-Institute, Hamburg, Germany). pGL3-basic derived luciferase reporter vectors, pSP1, pSP2, pCP and pXP, were previously generated and described^17^. The CREB5 expression vector pcDNA3.1/V5-CREB5, the luciferase reporters FXRα promoter pGL3-FXRp, and wildtype and mutated pmiR-CREB5-3′UTR were constructed, and luciferase reporter assays were performed as described in the Supporting Materials and Methods.

### Cell proliferation and cell cycle analysis

Cell proliferation was measured using WST-1 Cell Proliferation Reagent Kit I (Roche Diagnostics) and a previously described ^3^H-thymidine incorporation assay^17^. For cell cycle analysis, HepG2.2.15 cells were transfected with 20 nM of miRNA or control miRNA (miR-con), cultured for 48 h, treated with or without 4 μg/ml of aphidicolin or 100 nM of nocodazole for an additional 24 h and fixed in the presence of 70% ethanol at 4 °C. After washing, the fixed cells were incubated in PBS containing 20 μg/ml of propidium iodide, 200 μg/ml RNase A, and 0.1% Triton X-100 (BD Biosciences, Bedford, MA) at 37 °C for 20 min. The stained cells were then analyzed for cell cycle distribution using FACScaliber flow cytometer (BD Biosciences).

### Microarray analysis

Total RNA was isolated from HepG2.2.15 cells transfected with miR-449a and miR-con and subjected to microarray analysis using Affymetrix Human Genome U133A Plus 2.0 Array according to the manufacturer’s instructions. Differentially expressed genes were identified using Student’ *t* test on log-transformed data, and the results are represented as a heatmap using Spotfire (TIBCO Software Inc., Somerville, MA). These genes were further subjected to Gene Set Enrichment Analysis (GSEA) to identify the biological patterns of the genes. The significance threshold for the permutation test was set at P < 0.05.

### Statistical analysis

The statistical analysis was carried out using GraphPad (GraphPad Software Inc., San Diego, CA). Analysis of variance with Student’s t-test was used to determine significant differences in multiple comparisons. P < 0.05 was considered to be statistically significant. Representative data from a series of at least three experiments are shown; data are presented as the means ± standard deviation (STD).

## Additional Information

**How to cite this article**: Zhang, X. *et al.* Epigenetically regulated miR-449a enhances hepatitis B virus replication by targeting cAMP-responsive element binding protein 5 and modulating hepatocytes phenotype. *Sci. Rep.* **6**, 25389; doi: 10.1038/srep25389 (2016).

## Supplementary Material

Supplementary Information
