# Supplementary material for: Epigenetically regulated miR-449a enhances hepatitis B virus replication by targeting cAMP-responsive element binding protein 5 and modulating hepatocytes phenotype
Source: Sci Rep. 2016 May 3;6:25389. doi: 10.1038/srep25389 (PMC4853741; doi:10.1038/srep25389)
Supplement: Supplementary Information [file srep25389-s1.pdf]

Supplementary information to:

Epigenetically regulated miR-449a enhances hepatitis B virus replication by targeting cAMP-responsive element binding protein 5 and modulating hepatocytes phenotype

Xiaoyong Zhang, Hongyan Liu, Zhanglian Xie, Wangyu Deng, Chunchen Wu, Bo Qin, Jinlin Hou, Mengji Lu*

Table of contents:

Supporting Materials and Methods

Vectors construction--2
Luciferase reporter assay---3

Supplemental Tables

Supplemental Table 1---4
Supplemental Table 2---5

Supplemental Figures

Suppl. Fig. 1-- 6
Suppl. Fig. 2-- 7
Suppl. Fig. 3---8
Suppl. Fig. 4---9-10
Suppl. Fig. 5--11-12
Suppl. Fig. 6--13-14
Suppl. Fig. 7---14

Supporting Materials and Methods

Vectors construction

The full-length coding region for human CREB5 was amplified by RT-PCR on the total RNA isolated from HepG2.2.15 cells. The RT-PCR product was cloned into pcDNATM3.1/V5-His TOPO vector (Invitrogen) to generate CREB5 expression vector pcDNA3.1/V5-CREB5. The 3'UTR of CREB5 which including both miR-449a binding sites was amplified and cloned downstream of the firefly luciferase gene of pMIR-REPORT vector by restriction enzymes MluI and HindIII (Ambion, Austin, TX). The firefly luciferase reporter pmiR-CREB5-3UTR with two mutated FXR binding sites was created using the QuickChange Site-Directed Mutagenesis Kit (Stratagene, La Jolla, CA). The Mut1 mutation converted the sequence located between nt 3259 and 3266 from 5-ACACTGCC-3 to 5-GAGAGCAG-3. The Mut2 mutation converted the sequence located between nt 3318 and 3325 from 5-CACTGCCA-3 to 5-AGAGTTAG-3. The 5'-flanking genomic sequence (approx. 1.8 kb) of FXRα gene was amplified from genomic DNA of HepG2.2.15 cells. The PCR products were digested and inserted between MluI and BglII restriction sites into pGL3-basic vector (Promega, Madison, WI), resulting in the luciferase reporter vector pGL3-FXRp.

All clones were subjected to DNA sequencing to verify the correctness of the nucleotide sequences. The primers used for cloning are listed in supplementary table 2 (synthesized by Biomers, Ulm, Germany).

Luciferase reporter assay

HepG2.2.15 or Huh7 cells were seeded in 24-well plates and cultured for 24 h. 20 nM of miR-449a or miR-con was co-transfected with 100 ng of various firefly reporter and 100 ng of Renilla luciferase reporter TK-pRL (Clontech, Mountain View, CA) using Lipofectamine 2000. After 48 h, cells were washed with PBS and resuspended in lysis buffer of DualGlo Luciferase Assay system (Promega), followed by detection of luciferase activity in a luminescence reporter gene assay system according to the manufacturer's instructions (PerkinElmer, Norwalk, CT). All experiments were performed in triplicate, and the results presented are means of three separate experiments.

Supplemental Tables

Supplemental Table 1 miRNAs/siRNAs oligonucleotide sequences

Name	Type	Sequence 5'-3'
miR-con	miRNA control	UCACAACCUCCUAGAAAGAGUAGA
miR-1	Mature sequence	UGGAAUGUAAAGAAGUAUGUAU
miR-21	Mature sequence	UAGCUUAUCAGACUGAUGUUGA
miR-26a	Mature sequence	UUCAAGUAAUCCAGGAUAGGCU
miR-29b	Mature sequence	UAGCACCAUUUGAAAUCAGUGUU
miR-34a	Mature sequence	UGGCAGUGUCUUAGCUGGUUGU
miR-195	Mature sequence	UAGCAGCACAGAAAUAUUGGC
miR-222	Mature sequence	AGCUACAUCUGGCUACUGGGU
miR-224	Mature sequence	CAAGUCACUAGUGGUUCCGUU
miR-449a	Mature sequence	UGGCAGUGUAUUGUUAGCUGGU
miR-532-5p	Mature sequence	CAUGCCUUGAGUGUAGGACCGU
siFXRA	targeting sequence	CCGACTTATCCTAATGCGAAA
siMMAB	targeting sequence	CTGATAGATCCTGCTGTCTCA
siGAL	targeting sequence	CAGGTCATTCAGCGACAAGAA
siDDI2	targeting sequence	CCAGTGCAGTTCCCAAACTTA
siTMEM194B	targeting sequence	CAGAATTGTATATATCGCAGA
siCREB5	targeting sequence	AACAGTATTCTGTAGGATCTA
siMCM10	targeting sequence	ACGGCGACGGTGAATCTTATA
siSASS6	targeting sequence	TTGGACAGTCTCTTCGAATTA
siMYBL1	targeting sequence	AGCCATGGAATGCCAATTTAA
siZMAT1	targeting sequence	ACCAACGACCATACCATATTT
siAPOBEC3B	targeting sequence	AAGCAATGTGCTCCTGATCAA

All the human mature miRNA mimics and siRNAs were purchased from Qiagen Company (Hilden, Germany). The miRNA and siRNA nonspecific negative control (miR-con and siR-con) were purchased from Dharmacon (miR-con, miRIDIAN microRNA Mimic Negative Control #1, Cat. CN-001000-01; siR-con, siGENOME Non-Targeting siRNA #2; Cat. D-001210-02. Lafayette, CO).

Supplemental Table 2 Primers used for real time PCR and luciferase reporter cloning

Gene name	Application	Type	Sequence 5'-3'	Position of 5'-base
HBV DNA	real time PCR	forward	GTTGCCCGTTTGTCCTCTAATTC	465
		reverse	GGAGGGATACATAGAGGTTCCTT	563
HBV RNA	real time RT-PCR	forward	CCGTCTGTGCCTTCTCATCT	1551
		reverse	TAATCTCCTCCCCCAACTCC	1756
CREB5	CREB5 cloning	forward	TTGGTGACTGCAGGAAGC	344
		reverse	AAGAATCGGATTCAGGTCTGTTC	1914
CREB5 3UTR	pmiR-CREB5-3UTR	forward	CGACGCGT TACTCCATTCTCCTCCCTCAGCC	3151
		reverse	CCTAGATCT CTTCTCCTTGGCATTCTTGTAGTAG	4926
FXRα promoter	pGL3-FXRα-promoter	forward	CGACGCGT TGGAGGATGCACCATA	-1663
		reverse	CCTAGATCT CAAGGCCCTGGGAGGA	129

The underlined parts of primers indicate the specific cleavage sites of restriction enzymes ("_",MluI ; "_ _", BglII; "….''HindIII).
Reference Genebank accession No.: HBV, V01460; CREB5, NM_182898; FXRα, U68233

Supplemental figures

Suppl. Fig. 1

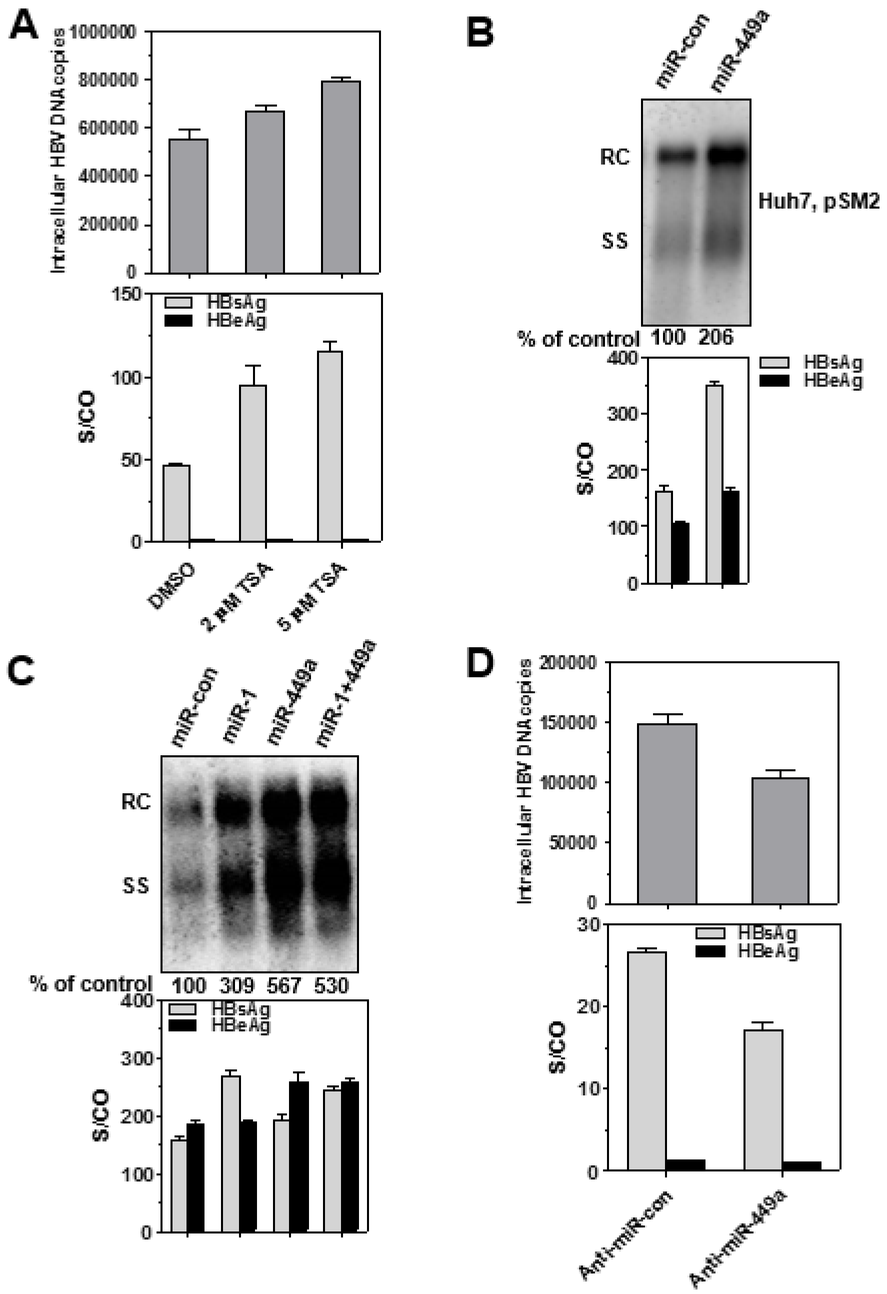

Suppl. Fig. 1 Regulation of HBV replication and antigen expression by miR-449a and its inhibitor anti-miR-449a. (A) HBV plasmid pSM2-transfected HL-7702 cells were treated with 2 or 5 μM of TSA for 24 hours, and intracellular HBV DNA was determined by realtime PCR using HBV specific primers. The levels of secreted

HBsAg and HBeAg in culture media were measured by the CMIA test. (B) The HBV plasmid pSM2 was co-transfected with 20 nM of miR-449a in Huh7 cells for 3 days, and HBV replication was determined by Southern blotting. The levels of secreted HBsAg and HBeAg in culture media were measured by the CMIA test. (B) HepG2.2.15 cells were transfected with 20 nM of miR-1, miR-449a or both miRNAs for 4 days, and HBV replication was determined by Southern blotting. The levels of secreted HBsAg and HBeAg in culture media were measured by the CMIA test. (D)The HBV plasmid pSM2 was co-transfected with 50 nM of Anti-miR-449a in Huh7 cells for 3 days, and HBV replication was determined by realtime PCR. The levels of secreted HBsAg and HBeAg in culture media were measured by the CMIA test.

Suppl. Fig. 2

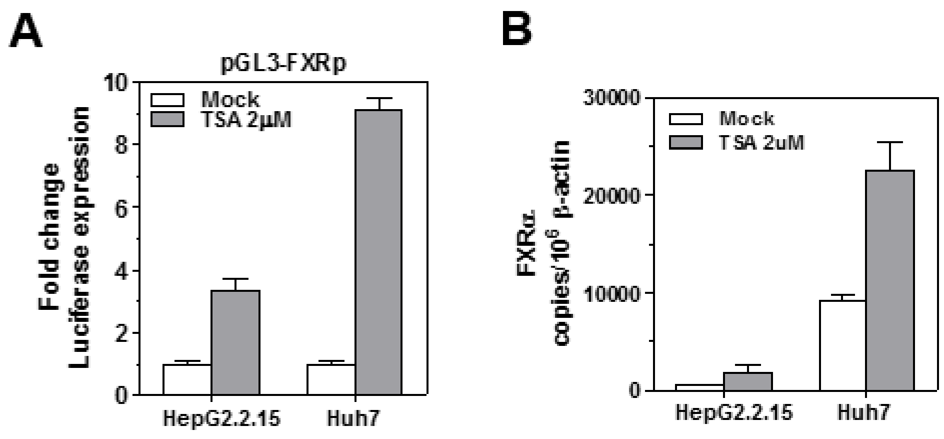

Suppl. Fig. 2 Regulation of FXRα promoter activity and RNA expression by TSA treatment. (A) A firefly luciferase reporter pGL3-FXRp (100 ng) containing the FXRα promoter region and the Renilla luciferase reporter TK-pRL (100 ng) were transfected into HepG2.2.15 cells or Huh7 cells. Luciferase activity was measured

after treatment with 2 μM of TSA for 24 h and normalized against a mock treatment. (B) HepG2.2.15 cells and Huh7 cells were treated with 2 μM of TSA for 24 h, and FXRα mRNA expression was measured by real-time RT-PCR.

Suppl. Fig. 3

A

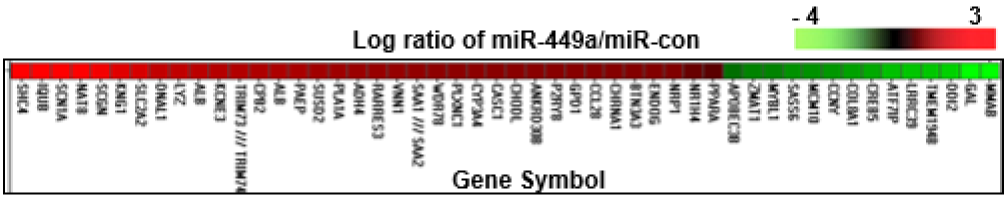

B

miR-449a binding sites	predicted consequential pairing of target region (top) and miRNA (bottom)	seed match
Position 3317-3323 of CREB5 3' UTR<br>hsa-miR-449a	5' ...AUCUGUCUCUGAAGUCACUGCCA...<br>||||||| 3' UGGUCGAUUGUUAUGUGACGGU	8mer
Position 3259-3265 of CREB5 3' UTR<br>hsa-miR-449a	5' ...GGGAAAAUCCUGUCACACUGCCU...<br>||||||| 3' UGGUCGAUUGUUAUGUGACGGU	7mer-m8

Suppl. Fig. 3 Identification of miR-449a regulation of FXRα and CREB5 expression. (A) Microarray analysis of differentially regulated cellular genes in HepG2.2.15 cells after transfection with 20 nM of miR-449a or miR-con for 4 days. The gene expression profiles of miR-449a- and miR-con-transfected cells were compared, and 50 differentially regulated genes with an increase of approximately 2.0-fold or with a reduction of ≥ 50% were selected. A heatmap for the selected genes was generated using Spotfire according to the log ratio of fold changes. (B) Sequence alignment of the miR-449a seed sequence and its conserved target site in the 3'UTR of CREB5 according to Targetscan software.

Suppl. Fig. 4

A

GSEA Results Summary
(miR-449a VS miR-con)

GeneSet name	CACTGCC,MIR-34A,MIR-34C,MIR-449
Downregulated in class	miR-449a
Enrichment Score (ES)	-0.3399606
Normalized Enrichment Score (NES)	-1.4754347
Nominal p-value	0.0
FDR q-value	0.2869454
FWER p-Value	0.901

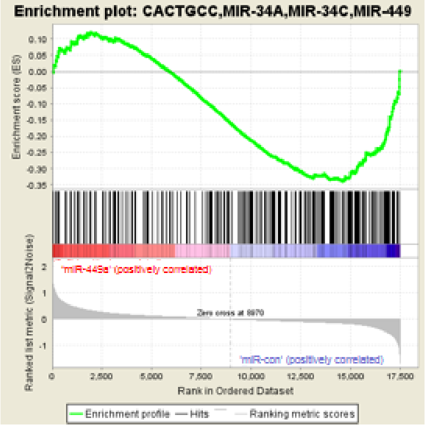

B

GSEA Results Summary
(miR-449a VS miR-con)

GeneSet name	Cell_CYCLE_PHASE
Downregulated in class	miR-449a
Enrichment Score (ES)	-0.4690911
Normalized Enrichment Score (NES)	-1.6696345
Nominal p-value	0.0
FDR q-value	0.62125076
FWER p-Value	0.867

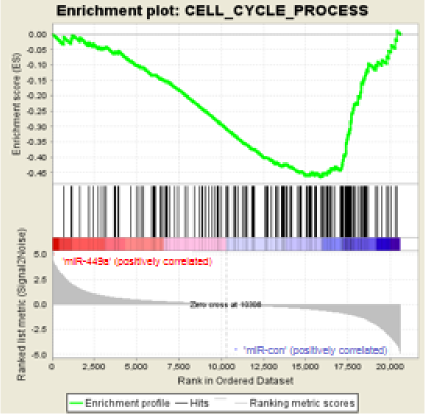

C

GSEA Results Summary
(miR-449a VS miR-con)

GeneSet name	HSIAO_LIVER_SPECIFIC_GENES
Upregulated in class	miR-449a
Enrichment Score (ES)	-0.39319417
Normalized Enrichment Score (NES)	1.5520793
Nominal p-value	0.0
FDR q-value	1
FWER p-Value	1

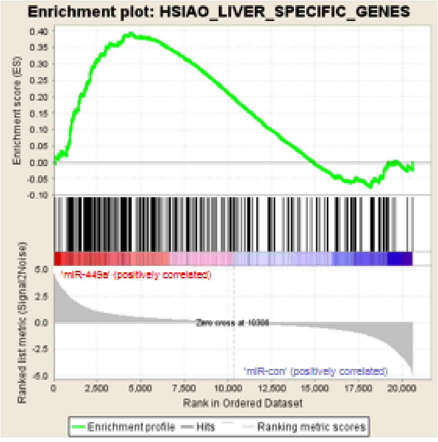

Suppl. Fig. 4 GSEA enrichment plot of differentially regulated genes in miR-449a-transfected HepG2.2.15 cells. Microarray analysis of differentially regulated genes in HepG2.2.15 cells after transfection with 20 nM of miR-449a or

miR-con for 4 days. These genes were further analyzed by GSEA using the Molecular Signature Database (MSigDB). miR-449a target genes (A) and cell cycle regulation genes (B) were significantly downregulated in the miR-449a group, whereas liver-specific genes were up-regulated. Vertical bars along the *x* axis of the GSEA plot denote the positions, within the ranked list, of genes in a given set. ($P < 0.001$).

Suppl. Fig. 5

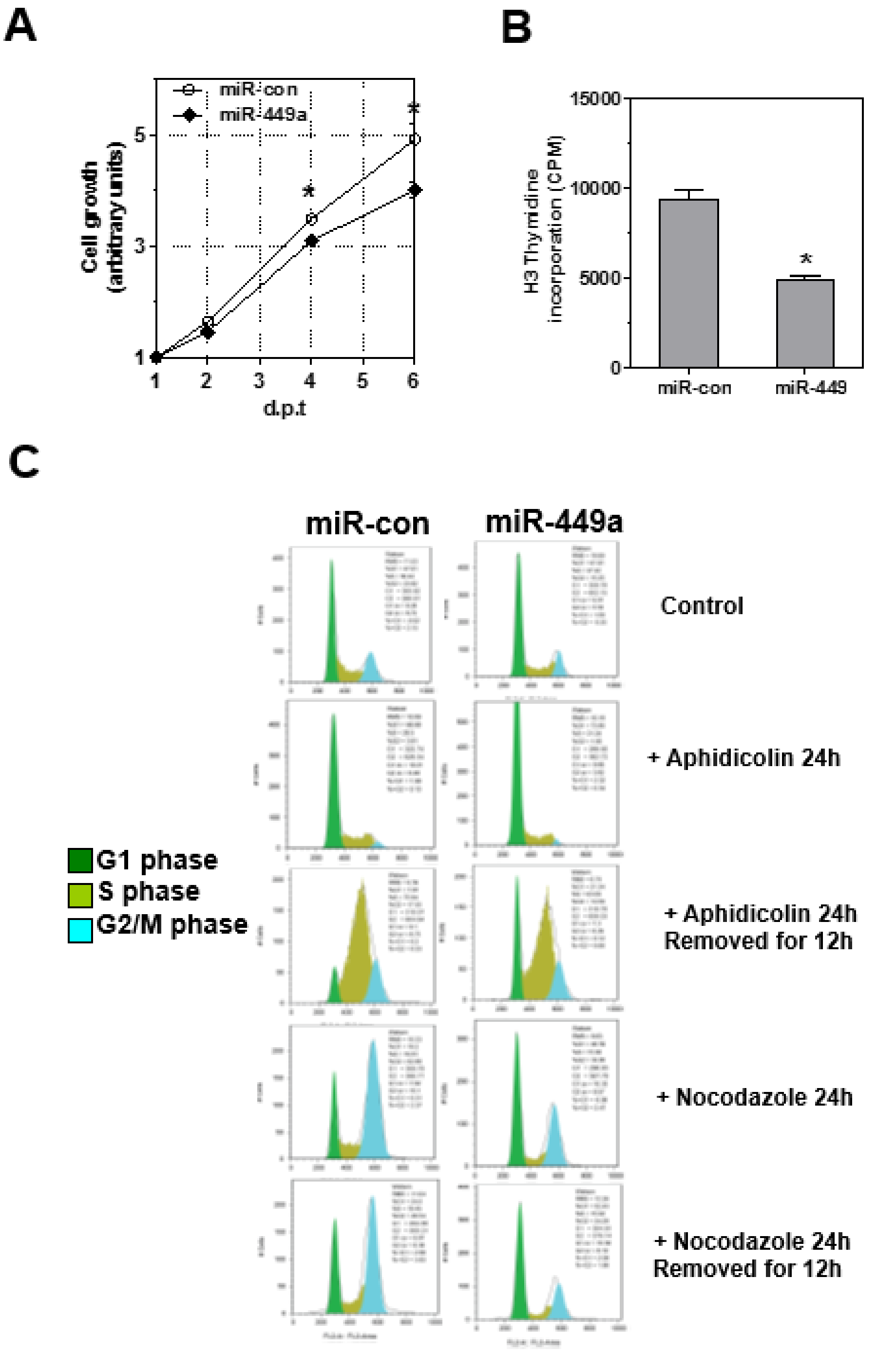

Suppl. Fig. 5 miR-449a inhibits cell proliferation and arrests the cell cycle in Huh7 cells. Huh7 cells were transfected with 20 nM miR-449a or miR-con. After 24 h, the cells were split to perform the following experiments. (A) Cells were distributed into 96-well plates at a density of 10,000/well, and cell growth was measured every

48 h for 6 days using a WST-1 assay. (B) Cells at a density of 10,000/well were serum-starved overnight for 48 h, followed by the addition of serum and ^{3}H-thymidine. Incorporation of ^{3}H-thymidine into cellular DNA after 4 h was measured using a scintillation counter. Each experiment was performed in triplicate with two different batches of transfected cells. (C) Cells were treated with nocodazole (100 nM), aphidicolin (4 μg/ml) or a medium control for 24 h. The distribution of cells in cell cycle phases G1, S, and G2/M was assessed by flow cytometry using propidium iodide staining. Representative histograms are displayed. *, $P < 0.05$.

Suppl. Fig. 6

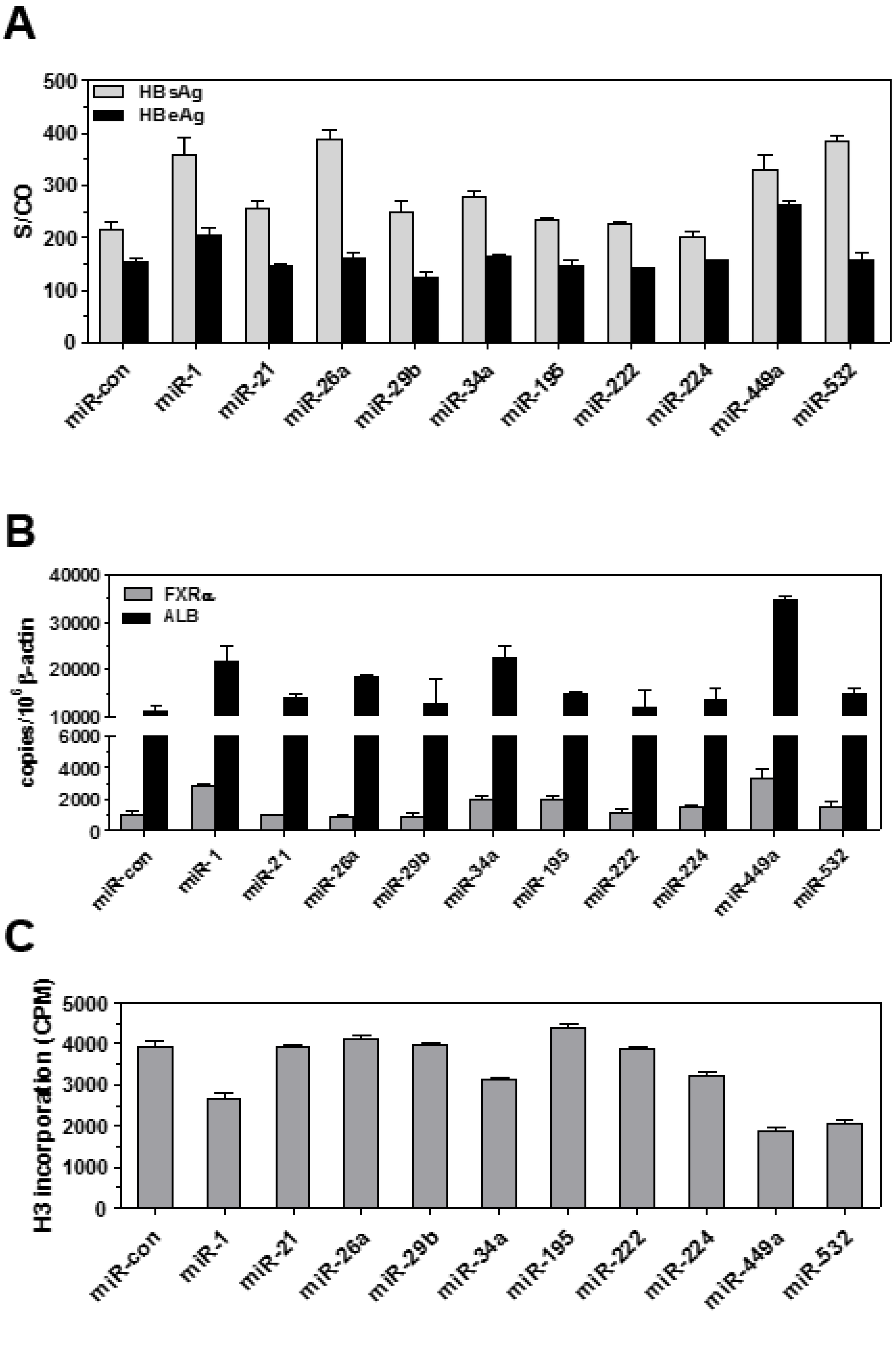

Suppl. Fig. 6 Regulation of HBV antigen expression, FXRα and ALB mRNA expression, and cell proliferation by HCC-related miRNAs. HepG2.2.15 cells were transfected with different miRNA mimics at 20 nM and cultured for 4 days. (A) The levels of secreted HBsAg and HBeAg in culture media were measured by the

CMIA test. (B) FXRα and ALB mRNA expression was determined by real-time RT-PCR. (C) Different miRNA-transfected cells at a density of 10,000/well were serum-starved overnight for 48 h, followed by the addition of serum and ^{3}H-thymidine. Incorporation of ^{3}H-thymidine into cellular DNA after 4 h was measured using a scintillation counter.

Suppl. Fig. 7

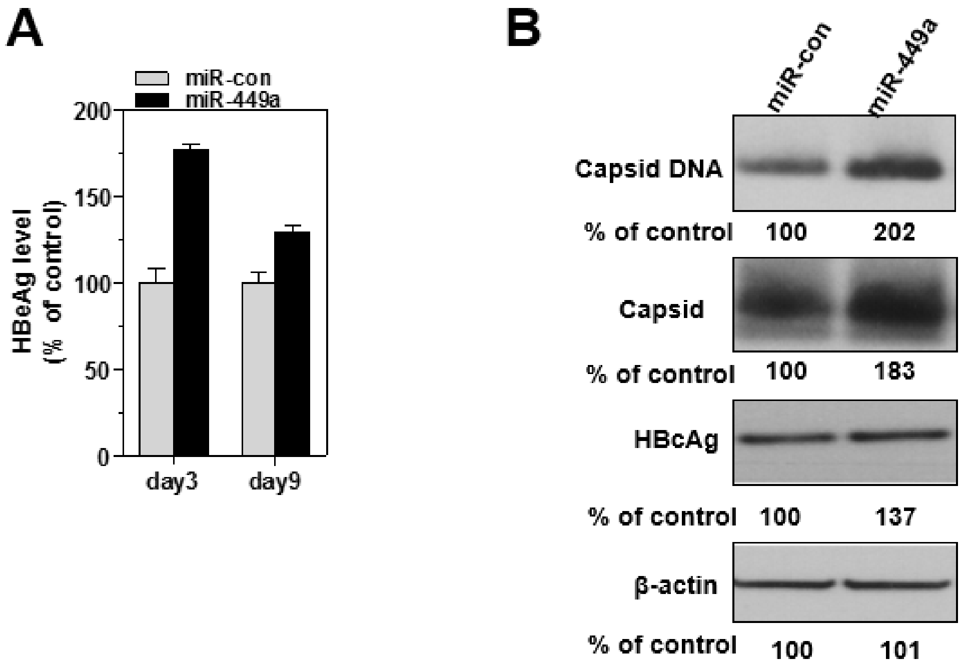

Suppl. Fig. 7 Effect of miR-449a on HBV gene expression in the cccDNA cell line HepDES19. HepDES19 cells were transfected with 20 nM of miR-449a or miR-con and maintained for 9 days. (A) The levels of HBeAg secreted into the culture medium were measured by the CMIA test at day 3 and 9. (B) HBV capsids containing DNA in the supernatant and intracellular HBcAg expression at day 9 were measured by Southern and western blotting, respectively.
